# The Effect of Oxygen Limitation on a Xylophagous Insect’s Heat Tolerance Is Influenced by Life-Stage Through Variation in Aerobic Scope and Respiratory Anatomy

**DOI:** 10.3389/fphys.2019.01426

**Published:** 2019-11-20

**Authors:** Marion Javal, Saskia Thomas, Philipp Lehmann, Madeleine G. Barton, Desmond E. Conlong, Anton Du Plessis, John S. Terblanche

**Affiliations:** ^1^Department of Conservation Ecology & Entomology, Faculty of AgriSciences, Centre for Invasion Biology, Stellenbosch University, Stellenbosch, South Africa; ^2^Department of Zoology, Stockholm University, Stockholm, Sweden; ^3^South African Sugarcane Research Institute, Mount Edgecombe, South Africa; ^4^CT Scanner Facility, Central Analytical Facilities, Stellenbosch University, Stellenbosch, South Africa; ^5^Physics Department, Stellenbosch University, Stellenbosch, South Africa

**Keywords:** *Cacosceles newmannii*, thermolimit respirometry, critical temperature, tracheal system, hypoxia

## Abstract

Temperature has a profound impact on insect fitness and performance via metabolic, enzymatic or chemical reaction rate effects. However, oxygen availability can interact with these thermal responses in complex and often poorly understood ways, especially in hypoxia-adapted species. Here we test the hypothesis that thermal limits are reduced under low oxygen availability – such as might happen when key life-stages reside within plants – but also extend this test to attempt to explain that the magnitude of the effect of hypoxia depends on variation in key respiration-related parameters such as aerobic scope and respiratory morphology. Using two life-stages of a xylophagous cerambycid beetle, *Cacosceles* (*Zelogenes*) *newmannii* we assessed oxygen-limitation effects on metabolic performance and thermal limits. We complement these physiological assessments with high-resolution 3D (micro-computed tomography scan) morphometry in both life-stages. Results showed that although larvae and adults have similar critical thermal maxima (CT_max_) under normoxia, hypoxia reduces metabolic rate in adults to a greater extent than it does in larvae, thus reducing aerobic scope in the former far more markedly. In separate experiments, we also show that adults defend a tracheal oxygen (critical) setpoint more consistently than do larvae, indicated by switching between discontinuous gas exchange cycles (DGC) and continuous respiratory patterns under experimentally manipulated oxygen levels. These effects can be explained by the fact that the volume of respiratory anatomy is positively correlated with body mass in adults but is apparently size-invariant in larvae. Thus, the two life-stages of *C. newmannii* display key differences in respiratory structure and function that can explain the magnitude of the effect of hypoxia on upper thermal limits.

## Introduction

Temperature is a key environmental driver of insect population dynamics since it directly affects life-history traits (Angilletta, 2009). In the context of climate change, geographic range shifts of insects are tightly linked with climate variability at a range of spatial and temporal scales (Rosenzweig et al., 2008). Understanding insect responses to temperature is therefore crucial in estimating how global warming will affect terrestrial arthropods (Bale et al., 2002; Huey et al., 2012). This is particularly true for insect pests, whose distribution and population dynamics are likely to be modified with changing climate (Battisti and Larsson, 2015; Lehmann et al., 2019a). For these reasons, estimating thermal limits is critical, since they represent the environmental limits of key traits, such as activity, survival, development and reproduction of ectothermic organisms (Angilletta, 2009). Yet thermal tolerances are affected by a range of methodological and environmental factors (Kovacevic et al., 2019) at diverse time-scales, from evolutionary to more recent thermal history. Thermal limits can be influenced, for example, by a large number of endogenous and exogenous parameters (Colinet and Boivin, 2011) including sex (Colinet and Hance, 2009; Nyamukondiwa and Terblanche, 2009), the rate of change in temperature (Kelty and Lee, 1999), acclimation or acclimatization history (Sejerkilde et al., 2003; Hoffmann et al., 2005; Koštál et al., 2011), reproductive status (Marshall and Sinclair, 2010; Javal et al., 2016), stage of development (Zhang et al., 2015), age (Bowler and Terblanche, 2008), body condition (Mitchell et al., 2017), humidity (Terblanche et al., 2011; Bubliy et al., 2012), or photoperiod (Kim and Song, 2000; Fischer et al., 2012).

One major factor of interest is atmospheric oxygen levels, and how this interacts with – or perhaps jointly determines – organismal thermal tolerance. For example, using a microarray approach, Boardman et al. (2018) recently found a novel candidate gene with unknown functions underlying high temperature stress resistance during hypoxia in *Drosophila melanogaster*. The “oxygen-and capacity-limited thermal tolerance” (OCLTT) hypothesis proposes to explain variation in heat tolerance in ectotherms under oxygen-limited conditions. This hypothesis states that insufficient capacity to supply oxygen to meet the oxygen demand of tissues can cause a progressive decline in animal performance as temperatures deviate from the optimum (Giomi et al., 2014). Many aquatic species conform to one or several predictions derived from the OCLTT hypothesis (Pörtner, 2010). The results for terrestrial insects, however, remain somewhat equivocal with some species showing strong support (Woods and Hill, 2004; Boardman and Terblanche, 2015), some showing partial support (Stevens et al., 2010) and others showing little or no support (Klok et al., 2004; Lehmann et al., 2019b; reviewed in Verberk et al., 2016b). This hypothesis has proven controversial and is the focus of several recent discussions questioning, in particular, the appropriate methods for falsifying predictions thereof (Clark et al., 2013; Jutfelt et al., 2014; Pörtner, 2014) and how widespread the evidence might be supporting OCLTT across diverse taxa (Verberk et al., 2016b; Jutfelt et al., 2018). In cases where strong or obvious support was not found for OCLTT in terrestrial insects, the reasons are typically unclear and not the focus of current investigation. Most commonly, structure-function dynamics of the respiratory system is widely expected to drive variation in support for the OCLTT hypothesis even in air-breathing insects (Verberk et al., 2016b). Differences in respiratory media (gas vs. liquid physico-chemistry, reviewed in Verberk et al., 2016a; Terblanche and Woods, 2018) may also account for variation in support for the OCLTT hypothesis among diverse arthropods.

The longhorned beetle *Cacosceles* (*Zelogenes*) *newmannii* Thomson (1877) (Coleoptera: Cerambycidae) is native to Mozambique, Eswatini and South Africa. Larvae of this beetle were found feeding on sugarcane (*Saccharum* sp. hybrids) for the first time in 2015 in the KwaZulu-Natal Province of South Africa. They dig galleries into the sugarcane stool and upwards into the stalks, causing considerable crop loss, as sucrose is stored in the lower nodes of mature sugarcane stalks. Although the level of hypoxia expected inside living plant tissues can vary with many different parameters (Pincebourde and Casas, 2016), this species is expected to show hypoxia adaptations, at least at the larval stage, owing to the wood-boring nature of larvae (Way et al., 2017), although it is possible the sugarcane plant is more aerated than the woody natural host plants in the region.

We explored the potential connection between thermal tolerance and oxygen limitation in this cerambycid beetle using diverse experimental laboratory approaches. We investigated whether oxygen availability influenced upper critical (=lethal) temperature through thermolimit respirometry (TLR; Lighton and Turner, 2004) in larvae and adults under normoxia and hypoxia. Our first prediction was that hypoxia reduced upper critical temperature (CT_max_) in both life stages. Our second prediction was that the magnitude of the oxygen limitation effect on CT_max_ would differ between adults and larvae, and that this was related to variation in respiratory structure and function. Adult beetles are expected to have greater aerobic scope (i.e., the excess capacity of the respiratory system to deliver oxygen) than larvae, because of more energetically demanding behaviors such as flight and/or mating. Adults are therefore expected to be able to maintain a more consistent respiratory pattern with decreasing oxygen level. Larvae on the other hand are expected to be more hypoxia-tolerant due to their saproxylic or wood-boring nature but to perhaps have limited aerobic scope since their activities do not imply large energy expenditure at this stage (e.g., they lack flight ability). Overall, these differences are expected to be associated with structural differences in the tracheal system (i.e., respiratory anatomy): in conditions of constant energy demand, adults would have a more developed tracheal system (estimated as e.g., total air volume) relative to their body size compared to larvae, indicative of greater potential upper capacity for oxygen flux (supply).

Using a combination of experimental respirometry and 3D-imaging of respiratory anatomy, we investigate functional hypoxia and temperature tolerance in a member of a previously unexamined group of terrestrial arthropods.

## Materials and Methods

### Experimental Animals

*Cacosceles newmannii* larvae and adults of both sexes were collected by hand on sugarcane farms in the Entumeni District (28° 55′S; 31°19′E), KwaZulu-Natal, South Africa (Way et al., 2017). Sampling of *C. newmannii* larvae included digging up sugarcane stalks, by prioritizing the ones looking unhealthy. Specimens were usually found in the base of the stalks, which had to be carefully split open to obtain the larvae. These were brought back to the laboratory at the South African Sugarcane Research Institute (SASRI) in 4 L plastic trays with perforated lids, containing moist peat in order to avoid stress or excessive desiccation. In the laboratory they were placed individually into 3 L plastic bottles with perforated lids, 30% filled with moist autoclaved peat, and a 22 cm long piece of sugarcane, with a 14 mm diameter hole bored 6 cm deep into one end to facilitate the larva’s entrance into the stalk to feed. The piece of sugarcane was replaced with fresh sugarcane every 3 weeks. The old stalk was carefully dissected to remove the feeding larva which was transfered into a new stalk. Larvae were then kept individually at 25°C in a 16L:8D regime, in an environmentally controlled room in the SASRI Insect Unit, before being couriered overnight, singly placed in plastic 250–600 ml jars filled with freshly crushed sugarcane, to the Department of Conservation Ecology and Entomology (ConsEnt), Stellenbosch University, Western Cape Province, South Africa. Adults were found flying (mainly males) or walking (mainly females) in sugarcane fields, mainly in January and February. They were collected by hand and transported individually in 250–600 ml plastic jars with perforated lids to ConsEnt. Shredded paper was placed in the bottle to provide a perching substrate and some protection from excessive movement for the adults, and a damp a piece of cotton to provide water in transit.

### Thermolimit Respirometry

Larvae and adults were placed individually in flow-through chambers to perform TLR. All individuals were weighed before and after respirometry on a microbalance (to 0.1 mg; AB104-S/Fact, Mettler Toledo International, Inc., Columbus, OH, United States), and the average value of the two measurements was used for statistical analyses. Thermal limits from metabolic rate data (V̇_CO__2_ CT_max_) under normoxia (21 kPa O_2_) and hypoxia (2.5 kPa O_2_, balance N_2_) were assessed following methods adapted from Boardman and Terblanche (2015). Briefly, air flow was regulated at 200 ml min^–1^ (STPD) [controlled by a mass flow control valve (SideTrak, Sierra International, United States)] into a Li-7000 infra-red CO_2_/H_2_O analyzer fully calibrated for CO_2_ at 386 ppm (balanced nitrogen) and water at 5°C dew point. The standard LiCor software (Li-Cor, Lincoln, NE, United States) recorded the CO_2_ production differentially (V̇_CO__2_) in ppm. Activity was monitored using an infra-red activity detector (AD-2, Sable Systems International, Las Vegas, NV, United States). Animals were given a 15-min equilibration period at 25°C, after which the temperature in the chamber was ramped up at a rate of 0.06°C min^–1^ to 55°C using a programmable circulating and heating water bath (CC410wl, Huber, Berching, Germany). Baseline recordings were taken before and after each run to correct for potential drift, which was typically negligible. Sample sizes for each treatment are given in Table 1. Oxygen availability levels were chosen according to what is commonly used in similar work on terrestrial arthropods (e.g., Van Voorhies, 2009; Basson and Terblanche, 2010; Stevens et al., 2010; Owings et al., 2014; Boardman and Terblanche, 2015; Boardman et al., 2015, 2016) and in order to mimic extreme environmental levels (Pincebourde and Casas, 2016) as well as to maximize insects’ responses over the time-scales examined here.

**TABLE 1:** Summary data for adults and larvae under hypoxia and normoxia assessed in *C. newmannii*, and results of statistical comparison between adults and larvae for all variables.

	**Gas treatment**	**Mean mass (g)**	**COV at 25°C**	**Max. metabolic rate over 60 s (ml h^–1^)**	**CT_max_ (C°)**	**Absolute aerobic scope (ml h^–1^)**	**m.s. Absolute aerobic scope (ml h^–1^ g^–1^)**	**Factorial aerobic scope**	**Q_10_ 25–35°C**	**Q_10_ 35–45°C**
Larvae	Normoxia	1.99 ± 1.01 (9)	0.90 ± 0.24 (8)	1.64 ± 1.55 (8)	46.00 ± 0.85 (8)	1.68 ± 1.53 (8)	1.19 ± 1.23 (8)	57.36 ± 30.58 (8)	1.78 ± 1.86 (8)	1.77 ± 1.23 (8)
	Hypoxia	2.61 ± 1.10 (13)	0.21 ± 0.21 (12)	2.16 ± 1.84 (11)	42.21 ± 1.57 (11)	1.83 ± 1.72 (11)	0.94 ± 0.70 (11)	4.83 ± 1.64 (11)	0.62 ± 0.25 (12)	1.67 ± 0.31 (12)
Adults	Normoxia	1.32 ± 0.51 (14)	0.69 ± 0.48 (12)	5.87 ± 3.35 (11)	45.55 ± 1.32 (10)	5.97 ± 2.56 (11)	6.17 ± 3.36 (11)	144.33 ± 102.18 (12)	2.41 ± 1.96 (12)	1.46 ± 1.03 (12)
	Hypoxia	1.13 ± 0.46 (8)	0.29 ± 0.18 (7)	2.11 ± 1.68 (7)	43.77 ± 1.90 (7)	1.72 ± 0.82 (7)	2.38 ± 1.48 (7)	33.23 ± 35.25 (7)	0.69 ± 0.36 (7)	1.48 ± 0.75 (7)
Adult vs. larva		***F*(1,37) = 14.888, *p* < 0.001**	*F*(1,34) = 0.1689, *p* = 0.68368	*F*(1,35) = 8.141, *p* < 0.005	*Z* = −1.067, *p* = 0.286	***F*(1,34) = 12.733, *p* < 0.001**	***F*(1,34) = 25.040, *p* < 0.001**	***F*(1,33) = 11.479, *p* = 0.002**	*F*(1,34) = 2.3074, *p* = 0.138	*F*(1,34) = 1.0287, *p* = 0.3176

*Means and standard error of the mean (SEM) are shown for mean mass, coefficient of variation (COV) of V̇_*CO2*_ at 25°C, CT_*max*_, absolute and factorial aerobic scopes and Q10 values over several temperature ranges. Sample sizes are given between brackets. m.s., mass specific; Max, maximum. Significantly different comparisons between adults and larvae appear in bold font.*

### Determination of Respiratory Patterns and Ambient Oxygen Effects

Assuming that discontinuous gas exchange cycles (DGC, i.e., showing a clear prolonged spiracle-closed phase) are undertaken to defend a particular tracheal respiratory pO_2_ setpoint of 4–5 kPa (as argued for moth pupae in Hetz and Bradley, 2005), we investigated whether respiration patterns (continuous or discontinuous gas exchange cycles) were affected by variation in ambient oxygen availability. For determination of respiratory patterns, the same methodology as described for TLR was used. Five adults and eight larvae were placed sequentially in individual chambers maintained at 25°C. Chambers were then measured for 40 min at each of five randomly assigned oxygen partial pressures (0, 2.5, 5, 8, 10.5 kPa O_2_, balance N_2_), starting and ending with a measurement at 20.9 kPa O_2_ and pattern-type classified according to spiracle behavior (openings and closings). The levels of oxygen partial pressure were chosen in order to target a wide range of hypoxia levels, and are similar to what is commonly found in the insect literature (e.g., Basson and Terblanche, 2010; Owings et al., 2014; Boardman et al., 2016).

### Respirometry Data Extraction and Analysis

Expedata (v. 1.9.10, Sable Systems International) was used to transform recorded CO_2_ values in ppm to V̇_CO__2_ in ml CO_2_/h, to correct for potential baseline drift for all respirometry files (TLR and respiratory patterns data), and to record activity (body movement) data.

In addition, minimum and maximum metabolic rates over 60 s, and mean CO_2_ were extracted from the normoxia data of the TLR using Expedata. Effects of life stage and mean mass on these values, as well as on normoxia CT_max_ (for the specimens used in the TLR experiment only), were computed using ANCOVAs.

Critical temperatures were assessed from the TLR data for normoxia and for hypoxia using Expedata 1.9.10 (Sable Systems International). The coefficient of variation (COV) at 25°C, CT_max_ (defined as the point at which spiracles switched from high to low variability and indicating that spiracle control was lost, reliably seen as a smooth curve of V̇_CO__2_, Supplementary Figure S1), absolute aerobic scope (i.e., the difference between resting and maximum metabolic rates) and Q_10_ values over different temperature ranges [as defined by Bělehrádek (1930), the factor by which a reaction rate increases for a 10°C increase in temperature] were computed according to Boardman and Terblanche (2015). Statistical analyses of the TLR data were conducted in Statistica (StatSoft Inc., Tulsa, OK, United States). Normality of data was checked and, when violated, non-parametric tests were used. Larval and adult mass differences between oxygen treatments were analyzed with *t*-tests, and comparisons between groups were made with ANOVAs or Mann–Whitney *U*-tests, followed by Tukey *post hoc* tests.

Expedata 1.9.10 (Sable Systems International) was used to visualize the effect of ambient oxygen on the proportion of individuals showing DGC.

### Computation of the Total Internal Air Volume of Respiratory Anatomy of Adults and Larvae

X-ray micro computed tomography (μCT) was performed with scan parameters optimized according to du Plessis et al. (2017). For these measurements, 4 adults (2 males and 2 females) and 4 larvae were selected based on their size, such that a wide spectrum of available morphologies was represented. All samples were scanned with identical settings in order to ensure direct comparison, specifically 120 kV and 220 μA for X-ray generation, magnification was set to achieve a voxel size of 30 μm, and each image was acquired in 131 ms, with averaging and skipping of images to enhance image quality. A total of 2200 step positions were used in one full rotation of the sample.

Images were segmented and analyzed in Volume Graphics VGSTUDIO MAX 3.1 software. The segmentation was achieved using a combination of image morphological operations, with slight modifications depending on the size of the sample. The main challenge was to achieve a closed surface including the whole tracheal system including the connected internal air cavities, while not including exterior air spaces, despite the entire system being open. The final result was manually checked and potential errors were corrected by modifying the segmentation. The segmentation process was manual but started with an automated surface determination function, guiding the selection of void spaces. The final segmented volume was checked manually to ensure no exterior air was included in the segmentation, and only interior air was included. A skin of two voxels all around the outside of the sample was then eroded, to leave only the interior of the sample including material and air. This was used to calculate internal air volumes. These were transformed into scaling relationships of air volume against body mass with fitted linear regression. Data fitted better to the regression without log scaling and therefore statistical testing was done on untransformed data.

For analyzing the scaling relationships between the traits, we used the general equation *Y* = *aM*^*b*^ where *Y* is the air volume, *a* is a scaling coefficient, *M* is the body mass, and *b* is the scaling value (Huxley, 1932; LaBarbera, 1989). The slopes, i.e., *b*, were derived by using ordinary least squares regression estimates and then compared to the commonly used theoretical scaling values 1, 0.67, and 0.75 (Chown et al., 2007). When comparing air volume to mass, the value 1 reflects isometry and is often used as a null hypothesis in anatomy scaling studies (LaBarbera, 1989). The value 0.67 is also isometry that reflects surface area to volume geometry (Kozłowski et al., 2003). The value 0.75 reflects isometry under fractal similarity of nutrient supply which also means that an organ grows less than body size (West et al., 1997, 1999). We used slope tests with a *t*-statistic computed as *t* = slope1-slope2/SE1 (following e.g., Chown et al., 2007). This approach assumes no variance in the theoretical slope value but employs the estimated slope and standard error of the empirical data (Zar, 1997). However, while scaling patterns of respiratory structures are expected to vary among tracheated arthropod species, they may also be highly variable across and within stages of the same species due to the discontinuous growth of the tracheal system and, in some cases, complex life histories (e.g., occurrence of diapause) (Helm and Davidowitz, 2013; Vogt and Dillon, 2013; Greenlee et al., 2014). Therefore, no specific allometry model or anatomical scaling hypothesis has been adequately developed yet regarding the scaling relationship of the tracheal volume in this study with body size to test, and we instead sought here to statistically compare the estimated scaling slopes (regression exponents) with other scaling values commonly reported in the literature across a wide range of species (Chown et al., 2007) and between the two main life-stages investigated.

Visualizations of segmented volume data were performed using isosurface views in VGSTUDIO MAX. Tracheal volumes were colored according to their local volume, which is equivalent to local diameter, using wall thickness analysis (sphere method, in mm). This function calculated the largest sphere that fitted in any given segmented region, and is usually applied to analysis of objects with varying wall thickness, but in this case the local thickness of the tracheal volumes were colored for visual clarity of large vs. small areas of connected volumes.

## Results

### Thermolimit Respirometry

**FIGURE 1:**
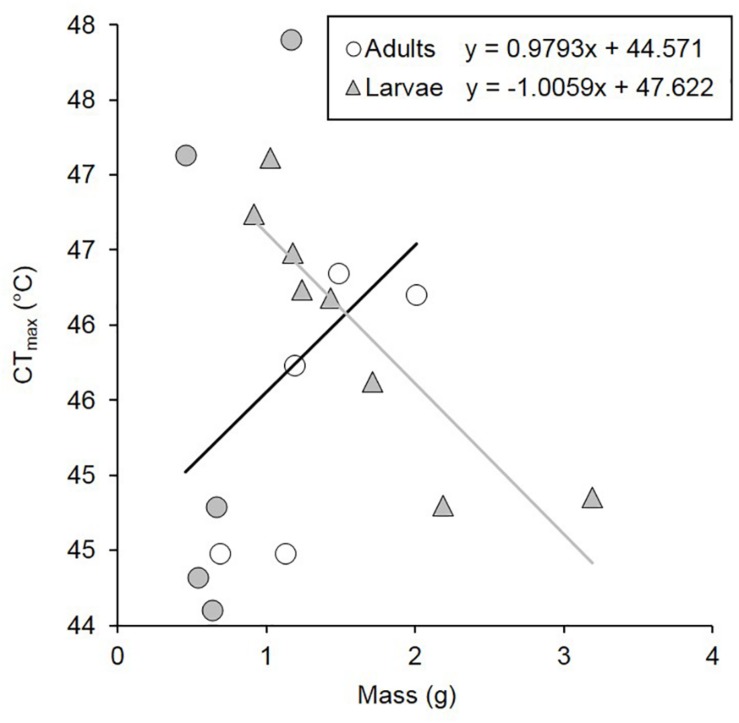
CT_max_ under normoxia of *C. newmannii* adults (males: white circles; females: gray circles) and larvae (gray triangles) of different masses. Regression equations are displayed on the figure (for adults, *r*^2^ = 0.135; for larvae, *r*^2^ = 0.811).

Larvae were significantly heavier than adults [*F*_(__1_,_37__)_ = 14.888, *p* < 0.001, Table 1]. Under normoxia, CT_max_ was significantly influenced by life-stage and by the interaction between life-stage and mean body mass (Table 2). CT_max_ increased with mass for adults (*R*^2^ = 0.135) but decreased with mass for larvae (*R*^2^ = 0.811) (Figure 1). Mass and life stage did not have any significant impact on mean CO_2_ production nor on minimum or maximum metabolic rates (Table 2). Normoxic metabolism of both life stages of this species was independent of body mass in our data.

**TABLE 2:** Results of factorial ANCOVAs with CT_max_ (A), minimum metabolic rate (Min MR) (B), maximum metabolic rate (Max MR) (C), and mean CO_2_ production at 25°C (D) of *C. newmannii* life stages at normoxia as dependant variables.

	**Effect**	**df**	**MS**	***F***	***p***
(A) CT_max_	**Life stage**	**1**	**7.031**	**6.823**	**0.020**
	Mean mass	1	0.001	0.001	0.976
	**Life stage × mean mass**	**1**	**5.589**	**5.424**	**0.035**
	Error	14	1.030		
(B) Min. MR	Life stage	1	0.056	0.527	0.474
	Mean mass	1	0.039	0.371	0.548
	Life stage × mean mass	1	0.001	0.006	0.941
	Error	26	0.106		
(C) Max. MR	Life stage	1	0.568	0.082	0.777
	Mean mass	1	15.081	2.165	0.153
	Life stage × mean mass	1	13.526	1.941	0.175
	Error	26	6.967		
(D) Mean CO_2_	Life stage	1	2.198	3.420	0.076
	Mean mass	1	0.006	0.010	0.921
	Life stage × mean mass	1	0.713	1.109	0.302
	Error	26	0.643		

*Significant results appear in bold.*

Individuals used for normoxia and hypoxia did not differ in mass (*t* = −1.26, *p* = 0.216 and *t* = 0.610, *p* = 0.550 for larvae and adults, respectively). Hypoxia lead to a significant decline in thermal tolerance for both larvae and adults: CT_max_ decreased by 3.8 and 1.8°C, respectively (Table 1).

### Variation in Metabolic Rates

Both mass-specific absolute aerobic scope and maximum metabolic rate were significantly higher for adults than for larvae regardless of the gas treatment [*F*_(__1_,_34__)_ = 25.040, *p* < 0.001 and *F*_(__1_,_34__)_ = 9.302, *p* = 0.004, respectively, Table 1]. The effect of hypoxia on maximum metabolic rate depended on life-stage: for larvae, hypoxia did not alter the maximum metabolic rate (Tables 1, 3 and Supplementary Figure S2), while in adults hypoxia decreased maximum metabolic rate to only 36% of normoxic levels (Tables 1, 3 and Supplementary Figure S2).

**TABLE 3:** Results of factorial ANOVAs with CT_max_ (A), maximum metabolic rate (B), and mass-specific absolute aerobic scope (C) of *C. newmannii* life stages as dependent variables, and the associated Tukey *post hoc* tests.

			**df**	**MS**	***F***	***p***	**Tukey *p***
(A) CT_max_ (°C)		Life stage	1	2.48	1.21	0.281	
		**Gas mixture**	**1**	**74.94**	**36.43**	**<0.001**	
	Life stage × gas mixture	Adult vs. larvae (normoxia)	1	5.79	2.81	0.104	0.97
		Adult vs. larvae (hypoxia)					0.25
		Normoxia vs. hypoxia (larvae)					**0.00**
		Normoxia vs. hypoxia (adults)					**0.03**
		Error	29	2.06			
(B) Maximum metabolic rate (mlCO_2_ h^–1^)		**Life stage**	**1**	**39.57**	**7.07**	**0.01**	
		Gas mixture	1	20.22	3.61	0.07	
	**Life stage × gas mixture**	Adult vs. larvae (normoxia)	**1**	**36.07**	**6.44**	**0.02**	**0.00**
		Adult vs. larvae (hypoxia)					0.99
		Normoxia vs. hypoxia (larvae)					0.96
		Normoxia vs. hypoxia (adults)					**0.02**
		Error	32	5.60			
(C) Absolute aerobic scope (mlCO_2_ h^–1^ g^–1^)		**Life stage**	**1**	**95.45**	**22.25**	**<0.001**	
		**Gas mixture**	**1**	**29.41**	**6.86**	**0.013**	
	**Life stage × gas mixture**	Adult vs. larvae (normoxia)	1	22.10	5.15	**0.03**	**0.00**
		Adult vs. larvae (hypoxia)					0.36
		Normoxia vs. hypoxia (larvae)					0.99
		Normoxia vs. hypoxia (adults)					**0.01**
		Error	32	4.29			

*Significant results are shown in bold.*

**FIGURE 2:**
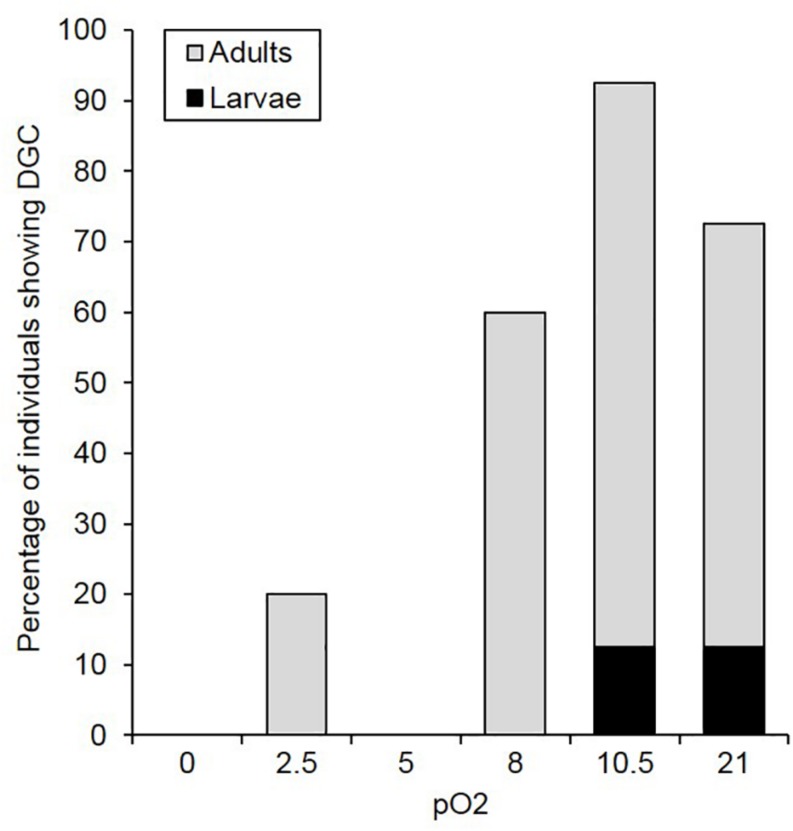
Percentage of *C. newmannii* individuals showing DGC as a function of oxygen concentration (pO_2_) for adults (light gray, *n* = 5) and larvae (black, *n* = 8).

Respiratory pattern switching was monitored at the individual level as a test of innate hypoxia sensitivity. Both life-stage and O_2_ partial pressure affected the proportion of individuals displaying DGC (life-stage: χ^2^ = 8.971, *p* = 0.003; O_2_ partial pressure: χ^2^ = 16.37, *p* < 0.001). More adults showed DGC than larvae across all O_2_ partial pressure treatments (Figure 2). Within life-stages, adult DGC proportion increased dramatically above 5 kPa O_2_. Only two larvae showed DGC, at 10.5 kPa O_2_ and at normoxia, the rest displayed continuous respiration patterns. At 0 and 2.5 kPa O_2_, V̇_CO__2_ was generally stable, in both adults and larvae, suggesting steady-state conditions had been achieved.

### Respiratory Anatomy

The entire respiratory structure was taken into account down to the minimum voxel resolution achieved in the CT scans. Smaller air spaces than the voxel size might not be included in the analysis, but this is expected to be negligible at the high resolution used here, given the large volume fraction of air space and the clearly visible tracheal systems in the datasets. In addition, the underestimation of the total tracheal volume due to the voxel size used in this study is similar for adults and larvae (Greco et al., 2014; Iwan et al., 2015), therefore allowing comparison of the two life stages.

**FIGURE 3:**
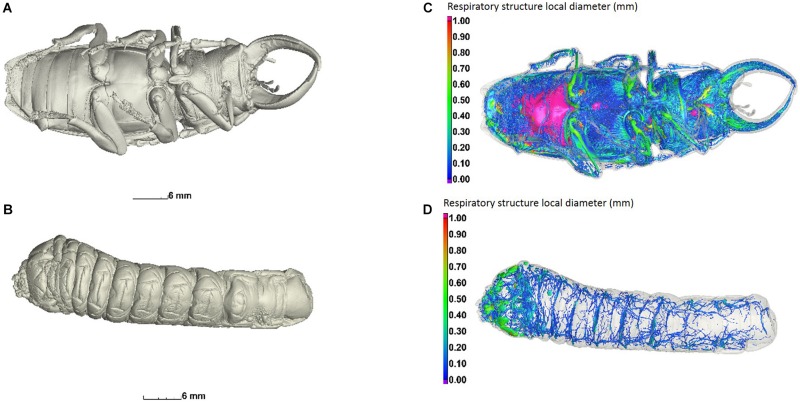
Central view of an adult male **(A)** and a larva **(B)** of *C. newmannii*. Ventral view of the tracheal structure of adult **(C)** and larva **(D)**. Air-filled structures are colored according to their local diameter, using wall thickness analysis (sphere method), and scaled as shown on the left. The wall thickness measures the size of the largest sphere that fits in every location inside the structure.

**FIGURE 4:**
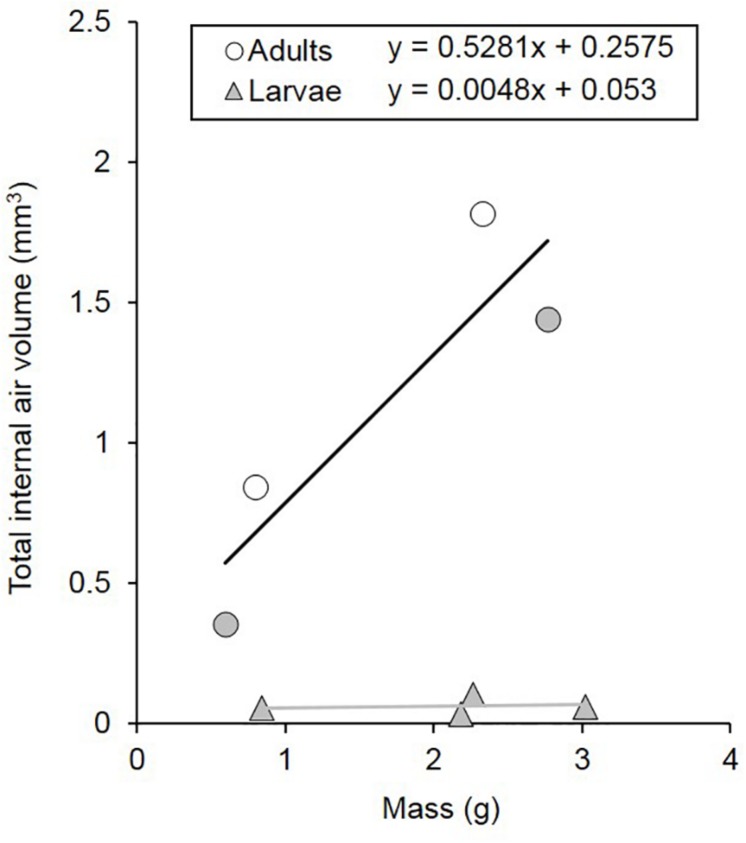
Tracheal volumes of *C. newmannii* adults (males: white circles, *n* = 2; females: gray circles, *n* = 2) and larvae (gray triangles, *n* = 4) of different masses. Regression equations are displayed on the figure (for adults, *r*^2^ = 0.791; for larvae, *r*^2^ = 0.021).

Total internal air volume was greater in adults than in larvae (*t* = 5.859, *p* = 0.009; Figures 3, 4). The internal air volume of adults increased with body mass (*R*^2^ = 0.791), while larval air volume remained constant even though body mass increased over threefold (*R*^2^ = 0.021) (Figure 4). However, even if the life-cycle and duration of each larval stage is not completely described yet for *C. newmannii*, individuals in this mass range are believed to belong to the same instar, according to preliminary observations. Neither larvae nor adults showed isometric growth (LaBarbera, 1989) between mass and respiratory structure air volume (*t* = 43.079, *p* < 0.001 and *t* = 2.456, *p* < 0.05, respectively), in both cases the scaling showed a negative allometry. We could not distinguish between scaling of 0.67 and 0.75 statistically for adults (*t* = 0.739, *p* > 0.25 and *t* = 1.155, *p* > 0.05, respectively). Interestingly, adults of both sexes showed a relatively large air sac in their abdomen, which was connected to the rest of the tracheal system (Figures 3A,C and Supplementary Figure S3).

## Discussion

In this study, we assessed the tolerance to different environmental conditions (oxygen and temperature) of a previously unreported cerambycid pest species, representing a taxonomic family which is often considered to be hypoxia-adapted over evolutionary timescales (Chappell and Rogowitz, 2000; Pincebourde and Casas, 2016). While many aspects of the specific biology of *C. newmannii* remain unknown (Way et al., 2017), here we found that the upper thermal tolerance (CT_max_) of this species is broadly consistent with estimates of CT_max_ in other Coleoptera from diverse families (Klok et al., 2004; Vorhees and Bradley, 2012; García-Robledo et al., 2016; Chidawanyika et al., 2017). *C. newmannii* larvae, it is presumed, typically live inside wood, a microenvironment that is assumed to be hypoxic and/or hypercapnic relative to the ambient atmosphere (Hoback and Stanley, 2001; Pincebourde and Casas, 2016). Regardless, it is frequently proposed that unlike adults, cerambycid larvae routinely experience hypoxia stress, as well as thermal constraints, given that behavioral thermoregulation is limited within plant tissues (Chappell and Rogowitz, 2000; Pincebourde and Casas, 2016; Javal et al., 2018). This microenvironment may therefore have contributed to evolved variation in larvae upper thermal limits. However, CT_max_ was not significantly different between larvae and adults in the present study, unlike a previous comparison of life-stages in a Lepidoptera species (Boardman and Terblanche, 2015).

One mechanistic theory proposed to explain variation in heat tolerance in ectotherms under hypoxic conditions is the OCLTT hypothesis. This hypothesis argues that insufficient capacity to supply oxygen to meet the oxygen demand of tissues can cause a progressive decline in animal performance as temperatures deviate from the optimum temperature (Giomi et al., 2014). For the OCLTT hypothesis to be supported, at least two key predictions can be made (reviewed in Verberk et al., 2016b). First, there should be a reduction in thermal performance, which is linked mechanistically with variation in thermal tolerance, when oxygen supply is reduced, and second, a decrease in the aerobic scope is expected when temperature approaches the critical limit (Verberk et al., 2016b). Even though many aquatic species conform to one or several predictions of the OCLTT hypothesis (Pörtner, 2010), the results for terrestrial insects remain equivocal and controversial (Verberk et al., 2016b). In our study, however, a reduction in CT_max_ was observed in both adults and larvae, although to different extents between the life-stages, when ambient oxygen supply was experimentally reduced, giving some support to the OCLTT hypothesis for a terrestrial arthropod.

If adults and larvae do not differ in terms of thermal tolerance, they do differ in several notable behaviors, as typically expected of holometabolous insect life-cycles, which in turn affects the way they can modulate their metabolism depending on their environmental constraints. Indeed, adults engage routinely in energy-costly behaviors such as fighting, flying or mating and were therefore expected to show a higher aerobic scope than larvae (Niven and Scharlemann, 2005). Larvae appeared to be more constrained in their aerobic scope than adults, but they were better able to sustain their maximum metabolic rate under hypoxic conditions. This could be related to the respiratory patterns that both life-stages can show. According to the metabolic rate hypothesis (Contreras and Bradley, 2009, 2010), insects with low metabolic rate will be more likely to show DGC than cyclic respiratory patterns. Since low metabolism is generally associated with low oxygen consumption (and CO_2_ production), spiracles can stay closed for longer without compromising the insect’s ability to meet its cellular metabolic demands. One can therefore assume that, as O_2_ partial pressure decreases, spiracles must remain open for a larger proportion of the time in order to meet a constant oxygen demand. This most likely explains why larvae maintain their metabolic rate at hypoxia but show a much lower proportion of DGC compared to adults, that are more likely to reduce their maximum metabolic rate to cope with hypoxic conditions. Indeed, hypoxia reduced maximum metabolic rate in adults to a greater degree than it did in larvae, thus reducing adults’ aerobic scope. A similar pattern was observed in two Heteroptera species where juveniles and adults did not differ in type of respiration and consequently did not differ in either CT_max_ nor the extent to which CT_max_ was influenced by oxygen levels (Verberk and Bilton, 2015).

The differences between life-stages observed in the present study could be due to ontogenetic variation or changes in respiratory structure-function relationships. Major remodeling or change in these relationships, within and across taxa, should produce predictable variation in the impacts of oxygen limitation on thermal tolerance. Depending on the type of metabolic theory and its mechanistic underpinnings (see e.g., Chown et al., 2007; Snelling et al., 2011a, b; Maino and Kearney, 2014; Harrison, 2018; Harrison et al., 2018) there may be considerable variation predicted in how the respiratory structure scales with body size. This scaling, in turn, is expected to have functional consequences for respiratory and athletic performance of a species (White et al., 2008; Snelling et al., 2011a, b). Differences among life-stages in breathing patterns and modulation thereof were expected to result from structural differences in the tracheal system. Indeed, in other insect species investigated to date (mainly Lepidoptera), the larval internal air volume stays constant within an instar, with increasing larval mass (Callier and Nijhout, 2011; Harrison, 2018). This unchanging internal air volume probably leads to an increasing mismatch between their resting metabolic rate and oxygen supply as the larvae grow, before molting (see e.g., Greenlee and Harrison, 2005 for Lepidopteran larvae, but see Kivelä et al., 2016, 2019), as suggested by the negative correlations between CT_max_ and body mass we observed. Adults, on the other hand, appear to have a larger tracheal system that increases with body mass and CT_max_. This result is unusual and perhaps noteworthy in air-breathing insects. One potential explanation for this outcome is that the respiratory system has most likely evolved for high oxygen demand activities (such as flight), and is therefore dramatically oversized for demands during resting periods (see discussions in Snelling et al., 2011a, b). Indeed, the presence of an air sac in the adults’ abdomen further supports such an assertion, even though flight muscles might also help in convection of air and the insect may still have limited capacity when it does not use these muscles (Komai, 2001). Such air sacs have been described for many other insect species (although typically in Orthoptera) in which they are intermittently compressed by contracted muscles during flight to create enhanced oxygen delivery to flight muscles (Wasserthal et al., 2018; reviewed in Chapman, 2012). In addition, since the respiratory pattern used by an insect can reflect its oxygen delivery capacity (Respiratory adequacy hypothesis; Contreras and Bradley, 2010), the enlarged oxygen delivery capacity of the adult tracheal system is a likely explanation for why adults are able to express DGC even under relatively low experimental O_2_ partial pressures.

In agreement with other intra-specific studies on insects (e.g., Lease et al., 2012; Harrison et al., 2014), our results imply that metabolic rate is size-independent for *C. newmannii*, at least in the uncontrolled, although mainly resting, state. Several biological explanations have been reviewed by Harrison et al. (2014) but none can be formally supported on the basis of our data. Beside biological hypotheses, this result can be attributed to the experimental respirometry protocol which did not seek to restrict or prevent activity of individuals; most animals were active during the equilibration period of the TLR trials. The absence of a relationship between normoxic metabolism and body mass could also be due to the relatively small sample size that was available, although this is likely offset by the large change in mass among specimens included (>fourfold difference in mass between the smallest and the largest specimens used, for both adults and larvae). However, detailed comparison of scaling relationships and the underlying mechanistic basis thereof was beyond the scope of this present study and would require far larger sample sizes before robust interpretations could be made.

To conclude, our study shows that marked divergence in terms of aerobic scope and respiratory patterns between life-stages were most likely due to both structural and functional differences, but these may be coupled with, or have co-evolved with, life-stage specific behavioral and physiological adaptations. Our data thus show that thermal limits, and any hypoxia effects thereon, are more predictable if respiratory structure-function relationships are fully characterized.

## Data Availability Statement

The datasets generated for this study can be found here: Thermolimit respirometry: 10.6084/m9.figshare.8263070, Internal air volume: 10.6084/m9.figshare.7392188.

## Author Contributions

JT conceived the project. DC co-ordinated the adult and larval field collection, laboratory preparation, and shipment. DC and JT secured the funding. ST, MB, and AD performed the experiments. MJ and PL ran analyses. MJ, PL, and JT co-wrote the manuscript. All authors revised and contributed to the final version of the manuscript.

## Conflict of Interest

The authors declare that the research was conducted in the absence of any commercial or financial relationships that could be construed as a potential conflict of interest.

## Supplementary Material

Supplementary Figure S3 can be found in FigShare at doi: 10.6084/m9.figshare.8397221

FIGURE S1 Example of thermolimit respirometry trace of a *C. newmannii* adult **(A,B)** and a larva **(C,D)** with carbon dioxide production (blue), temperature (red), and activity (green), under normoxia **(A,C)** and hypoxia **(B,D)**. The blue dotted arrows indicate CT_max_, which correspond to loss of spiracular control.

FIGURE S2 Mean plot of  CT_max_ **(A)**, and maximum metabolism **(B)**, for *C. newmannii* larvae (blue) and adults (green). Asterisks show significant differences among life stages.

FIGURE S3 Video illustrating the tracheal structures of a male adult *C. newmannii*. Air-filled structures are first colored according to their local volume, using wall thickness analysis (sphere method), and scaled as shown on the left, and then shown in black and white.
