# Supplementary figures and images for: The Effect of Oxygen Limitation on a Xylophagous Insect’s Heat Tolerance Is Influenced by Life-Stage Through Variation in Aerobic Scope and Respiratory Anatomy
Source: Front Physiol. 2019 Nov 20;10:1426. doi: 10.3389/fphys.2019.01426 (PMC6879455; doi:10.3389/fphys.2019.01426)

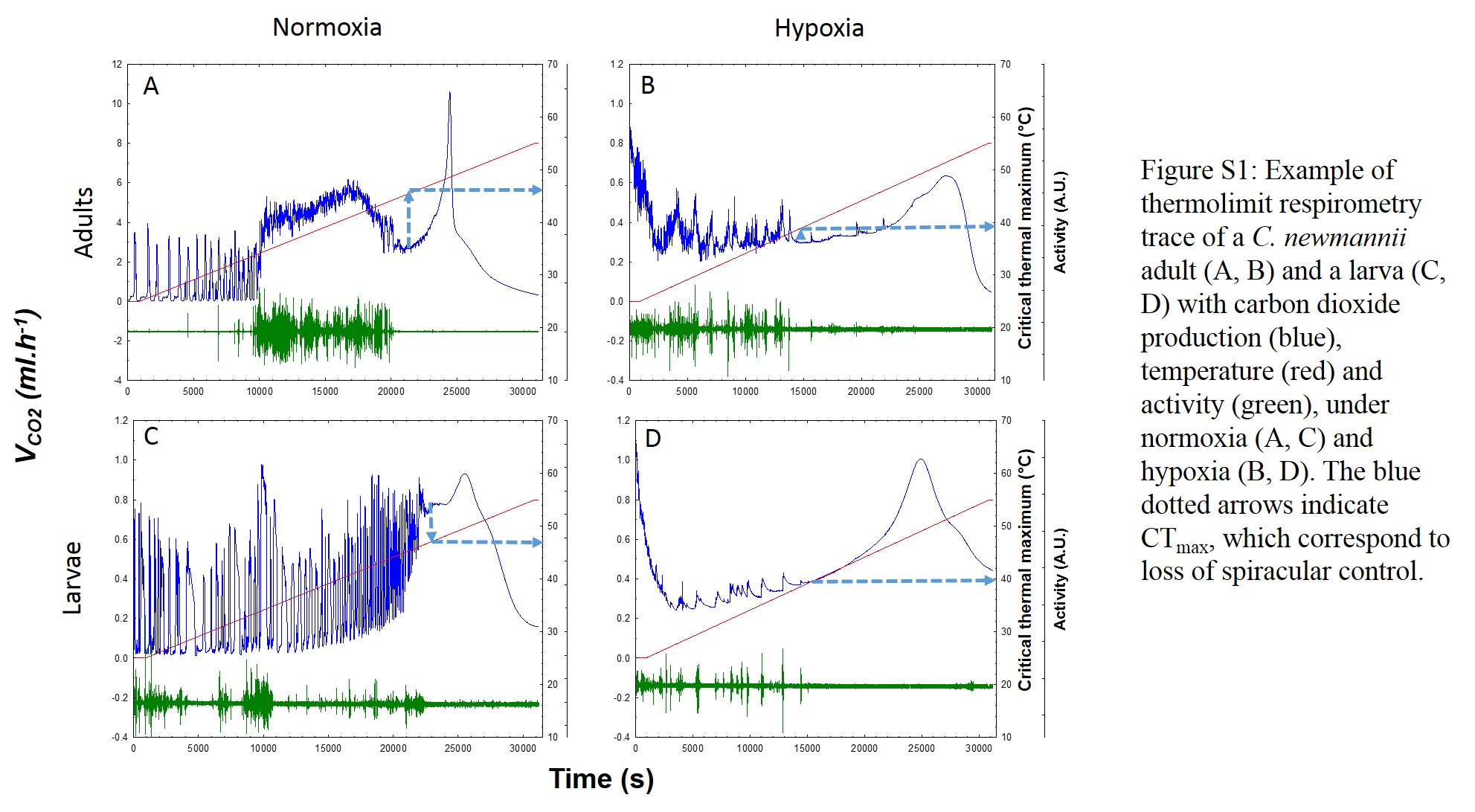

Supplement: FIGURE S1 — Example of thermolimit respirometry trace of a C. newmannii adult (A,B) and a larva (C,D) with carbon dioxide production (blue), temperature (red), and activity (green), under normoxia (A,C) and hypoxia (B,D). The blue dotted arrows indicate CTmax, which correspond to loss of spiracular control. [file Image_1.JPEG]
